# Superhydrophobic Substrates for Ultrahigh Encapsulation of Hydrophilic Drug into Controlled-Release Polyelectrolyte Complex Beads: Statistical Optimization and In Vivo Evaluation

**DOI:** 10.3390/pharmaceutics11060257

**Published:** 2019-06-01

**Authors:** Carol Yousry, Iman S. Ahmed, Maha M. Amin, Omaima N. El Gazayerly

**Affiliations:** 1 Department of Pharmaceutics and Industrial Pharmacy, Faculty of Pharmacy, Cairo University, Cairo 11562, Egypt; carol.yousry@pharma.cu.edu.eg (C.Y.); maha.amin@pharma.cu.edu.eg (M.M.A.); omaima.naim@pharma.cu.edu.eg (O.N.E.G.); 2 Department of Pharmaceutics & Pharmaceutical Technology, College of Pharmacy, University of Sharjah, Sharjah 27272, UAE

**Keywords:** superhydrophobic substrates, glass surfaces, chitosan, K-carrageenan, polyelectrolyte complex, in vivo absorption study

## Abstract

In this work, ultrahigh drug-loaded chitosan (Ch)/K-carrageenan (Kc) polyelectrolyte complex (PEC) beads were formed in situ by cross-linking in a glutaraldehyde-saturated atmosphere and were prepared on superhydrophobic substrates fabricated by spraying glass surfaces with ready-made spray for domestic use (NeverWet^®^). Verapamil hydrochloride (VP), a highly hydrophilic drug with a short biological half-life, was incorporated into a series of Ch-based and/or Ch/Kc-PEC-based beads to control its release profile in vivo. The formulation of VP-loaded beads was optimized using stepwise statistical designs based on a prespecified criterion. Several characteristics of the prepared beads, such as entrapment efficiency (EE%), in vitro drug release, swelling ratio, size and surface microstructure as well as molecular interactions between the drug and formulation ingredients, were investigated. In vivo pharmacokinetic (PK) studies were carried out using the rabbit model to study the ability of the optimized VP-loaded beads to control the absorption rate of VP. Results revealed that the prepared superhydrophobic substrates were able to fabricate VP-loaded beads with extremely high EE exceeding 90% *w*/*w* compared to only 27.80% when using conventional ionotropic gelation technique. PK results showed that the rate of VP absorption was well controlled following oral administration of the optimized beads to six rabbits compared to a marketed VP immediate release (IR) tablet, as evidenced by a 2.2-fold increase in mean residence time (MRT) and 5.24-fold extension in half value duration (HVD) over the marketed product without any observed reduction in the relative oral bioavailability.

## 1. Introduction

Controlled-release multiparticulate systems designed to deliver highly hydrophilic drugs with relatively short biological half-lives offer many therapeutic and clinical benefits due to reduction in dosing frequency. In addition, they are well known to be uniformly distributed in the gastrointestinal tract (GIT), resulting in more uniform absorption with no risk of dose dumping compared to single unit systems [1,2].

Hydrophilic drugs are generally difficult to encapsulate using conventional encapsulation techniques due to their rapid diffusion from the prepared systems to the external aqueous phase, which makes their formulation more challenging [3,4]. Attempts to overcome this challenge focused on modifying the external aqueous phase to a less favorable medium [5] by changing its pH and/or viscosity as well as by saturating it with an electrolyte to reduce the drug outflux [6].

Many of the encapsulation techniques proposed to produce polymeric particulate systems have limited applications due to the mandatory use of organic/toxic solvents or high temperature, difficulty in complete solvent removal in addition to low drug EE [7].

Inspired by the behavior of water droplets on the lotus leaf, many scientists have tried to mimic its ultra-hydrophobic surface. The combination of its hierarchical roughness with a low surface free energy is believed to be the main cause of the water repellency of this surface and its self-cleaning effect. Thus, scientists were able to fabricate superhydrophobic surfaces with high water contact angle (>150°) and low sliding angle (<10°) to be used in many life applications [8]. Although superhydrophobic substrates recently stepped into the field of pharmaceutical research due to their ease and suitability for the preparation of environmentally-friendly highly loaded polymeric beads, few studies have been carried out to widen such application.

Several studies reported the use of superhydrophobic surfaces to formulate beads from hydrophilic polymers with high EE. Upon application of the aqueous polymer solution to the superhydrophobic surface, it forms a spherical shape, which can be easily hardened under mild conditions without any need for an external liquid phase [4,9,10]. Song et al. [9] were the first to successfully encapsulate theophylline, as a model of highly water soluble molecule, into alginate beads using polystyrene superhydrophobic substrates treated with 1H,1H,2H,2H-perfluorodecyltrimethoxysilane. Lima et al. [10] used different substrates (polystyrene, aluminum and copper sheets) in the preparation of superhydrophobic surfaces to formulate temperature-responsive dextran-methacrylated/poly(*N*-isopropylacrylamide) beads for controlled release of encapsulated bioactive substances. Puga et al. [4] achieved high loading of 5-fluorouracil into pectin-coated chitosan microgels for oral administration using polystyrene superhydrophobic substrates. However, most of the drug was released within one hour except when using highly acidic dissolution medium (1% HCl), where the pectin-coated beads offered sustainment of drug release for almost 4 h. In all of these previous studies, authors assumed 100% EE of the active material inside the beads due to absence of an external liquid phase without considering possible drug diffusion to the superhydrophobic surface.

Hydrogels of natural polymers, such as chitosan (Ch), have been widely used in the preparation of controlled-release systems due to their biodegradability and biocompatibility [11]. Ch especially has both antacid and antiulcer activity, thus reducing drug irritation in stomach when taken orally. It is also considered as one of the highly basic polysaccharides that can absorb large amounts of water and swell, thus controlling drug diffusion and release [12]. Ch can be crosslinked with glutaraldehyde to form hard beads with controlled-release behavior. Polyelectrolyte complexes (PEC) of Ch with different anionic compounds (e.g., sodium alginate, sodium carboxymethyl cellulose, carrageenan, Carbopol, Eudragit) were reported to offer superior drug release controlling effect when formulating beads [13,14] or tablets [15,16,17].

**Figure 1:**
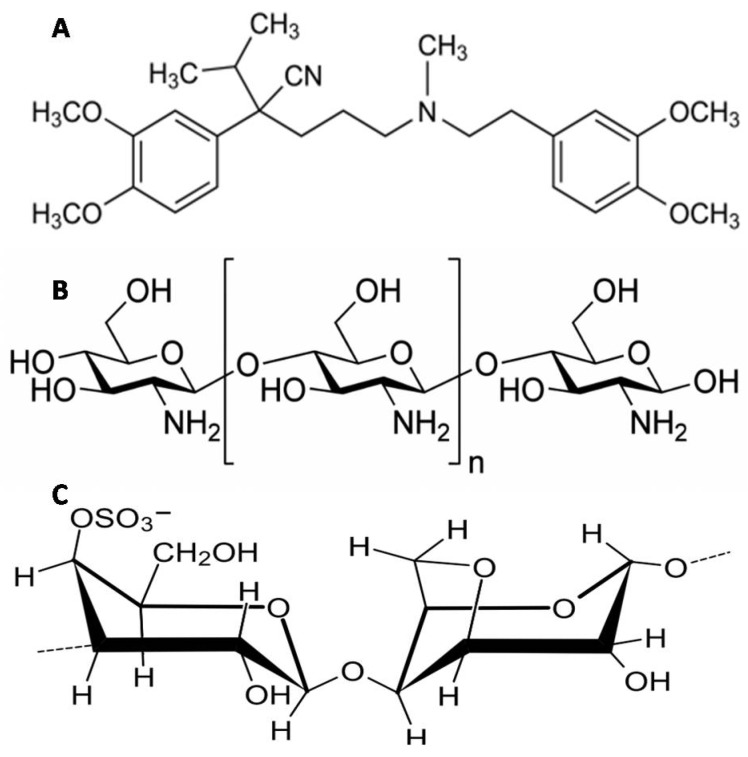
Chemical structures of (**A**) verapamil hydrochloride (VP); (**B**) Ch and (**C**) Kc.

Verapamil hydrochloride (VP, Figure 1A), the model drug used in this study, is a highly hydrophilic drug that suffers from poor oral bioavailability (20–30%) due to extensive pre-systemic metabolism in the liver and has a relatively short biological half-life (4 h) [18,19]. Several attempts were reported to overcome VP aqueous solubility and achieve a controlled-release behavior. Yassin et al. [1] were able to include only 42% of VP inside Ch gastroretentive beads [1,2]. In another study, composite Ch-transfersomal vesicles were formulated with VP EE% ranging from 24% to 64% [20]. Abdel Mouez et al. [21] reported that encapsulation of VP increases upon exclusion of the external phase when comparing VP encapsulation into microspheres using spray drying vs. precipitation techniques [20].

In an attempt to alter VP pharmacokinetic profile in vivo, superhydrophobic substrates were fabricated in this study and were utilized to prepare controlled-release ultrahigh VP-loaded Ch-beads and Ch/Kc-PEC-based beads. The formulated beads were prepared and hardened in a glutaraldehyde-saturated atmosphere to exclude the use of an external liquid phase in order to achieve maximum EE after tracking any drug diffusion to the superhydrophobic substrate. Ch (Figure 1B) was complexed with Kc (Figure 1C) to offer more controlled VP release from the beads. Two statistical designs were adopted to optimize the formulation conditions in order to achieve systems with highest EE% and controlled-release characteristics. Finally, in vivo study in rabbits was performed using selected optimized formulation, and pharmacokinetic (PK) results were compared to marketed immediate release VP tablets.

## 2. Materials and Methods

### 2.1. Materials

Glass slides (50 mm × 50 mm) were used in this study. NeverWet^®^ spray was purchased from Rust-Oleum (Vernon Hills, IL, USA). Chitosan low molecular weight (MW) (50–190 kDa), medium MW (190–310 kDa) and high MW (310–375 kDa) were purchased from Sigma-Aldrich Inc. Al. (St. Louis, MO, USA). Kappa-Carrageenan was purchased from Acros Organics (Morris County, NJ, USA). Verapamil HCl was supplied as a gift from El-Nasr Chemical Co. (Cairo, Egypt). Glutaraldehyde solution (50% *v*/*v*) and glacial acetic acid were purchased from El Nasr pharmaceutical chemicals company (Cairo, Egypt).

### 2.2. Preparation and Characterization of Superhydrophobic Substrates

Superhydrophobic substrates were prepared by applying “NeverWet^®^” spray on clean glass slides. Briefly, glass slides were thoroughly cleaned and dried, and then three thin layers of “base coat” were applied with 30 min intervals between each application. After complete drying of the base coat, the “top coat” was sprayed as another three layers with 5 min intervals, and then the glass slides were left to dry in the air before use. The roughness of the surface was observed by scanning electron microscopy. The static contact angles (CA) of water, olive oil, VP-Ch and VP-Ch/Kc dispersions were measured by dropping a 5 µL droplet of each of the above-mentioned liquids on the treated glass surface using contact angle meter DM-701 interfaced with KYOWA analysis system FAMAS software version 3.4 (KYOWA Interface Science Co., Ltd., Saitama, Japan) The same measurements were repeated on untreated glass surfaces to compare results. Sliding angles (SA) were measured for each of the above-mentioned liquids by slowly tilting the treated glass slide until the examined liquid droplet started to move. Results were recorded as the mean values of three sample measurements [22]. The fraction of the air in contact with the liquid droplet was also calculated from Cassie and Baxter’s equation [23] as follows,

cosθ_*_ = −1 + *f* (cosθ + 1) (1)

where θ_*_ and θ are the CA of the liquid droplet on the treated glass and untreated glass, respectively; *f* is the fraction of solid treated surface in contact with the liquid droplet and accordingly, (1-f) is the fraction of the trapped air beneath the liquid droplet.

### 2.3. Preparation of VP-Loaded Ch-Based Beads

A 3^2^ full factorial design was applied to evaluate the influence of the used Ch on the characteristics of the prepared beads. In this design, Ch molecular weight (MW) and concentration, each at three levels, were selected as the independent variables, whereas EE% and the rate of in vitro drug release from the prepared beads expressed as t_90%_ were evaluated as the dependent variables. Dispersions of different grades of Ch in 1% acetic acid solution were prepared at various concentrations according to the design shown in Table 1. Briefly, 80 mg of VP was added to Ch dispersions and thoroughly stirred using magnetic stirrer (SB162; Stuart, Staffordshire, UK) to ensure maximum solubilization of VP in the dispersions. For each dispersion, 15 µL droplets were placed on the prepared superhydrophobic glass surfaces. The glass slides were then gently placed on the top of a perforated rack inside a desiccator containing 250 mL of glutaraldehyde solution (50%, *v*/*v* in water) at its bottom as cross-linking agent without any contact with the glass slides. Thus, hardening of Ch beads was performed in a glutaraldehyde-saturated atmosphere to exclude the use of an external liquid phase and reduce drug leakage. Traces of un-reacted glutaraldehyde were removed under vacuum [4]. All nine formulations (F1–F9) were performed in a duplicate, randomized way to satisfy the statistical requirements. Furthermore, the values of the dependent variables were optimized with the optimization criterion set at the highest EE% and longest t_90%_ to yield the system with the highest desirability factor.

### 2.4. Preparation of VP-Loaded Ch/Kc-PEC-Based Beads

Polyelectrolyte complex (PEC) beads were prepared by complexing Ch with Kc to further prolong VP release from the beads [14]. Simply put, Kc (in different concentrations, 1.5%, 2%, 2.5%, 3%, 3.5%, 4%, 4.5% *w*/*v*) was dispersed in 1% *v*/*v* acetic acid solution containing 80 mg of VP (Table 2). The dispersions were then mixed with the optimized concentration and MW of Ch selected from the previous design (1.5% *w*/*v* and high MW respectively). The resultant dispersions were stirred thoroughly until homogenous dispersions were obtained [13,14]. For each dispersion, 15 µL droplets were placed on the prepared superhydrophobic surfaces and then transferred to glutaraldehyde atmosphere, as previously described. One factor design in a surface response study was applied to study the influence of Kc addition in various concentrations on the EE% and t_90%_ of VP using Design-Expert^®^ software, where the most desirable system was selected, as previously discussed, for further investigation.

### 2.5. Evaluation of VP-Loaded Beads

#### 2.5.1. Encapsulation Efficiency (EE%)

After complete solidification of the formulated beads, the superhydrophobic layer was peeled from the glass surface, immersed into 100 mL of water and stirred for 24 h to extract any residual amount of free un-encapsulated VP from the surface. On the other hand, the amount of the encapsulated drug was extracted with water for 24 h after complete crushing of the solidified beads. Extracted drug in water was measured using a UV Spectrophotometer (UV-1800; Shimadzu, Kyoto, Japan) at λ_max_ 278 nm [24]. A blank superhydrophobic layer was treated similarly to ensure the absence of any leaked materials that could interfere with the UV absorption at the pre-mentioned λ_max_. The EE% was calculated from the following equation,

(2) $$\mathrm{EE}\%=\frac{D_{e}}{D_{e}+D_{f}}\times 100$$

where D_e_ is the amount of VP encapsulated within the beads and D_f_ is the amount of free un-encapsulated VP.

#### 2.5.2. In Vitro Release Studies

The release of VP from the beads was determined in a dissolution tester (Varian, VK7000; Varian Inc., Cary, NC, USA) following the USP paddle method. All studies were done in 300 mL 0.1 N HCl (pH 1.2) for the first 2 h to simulate gastric conditions, then 100 mL of 0.2 M tribasic sodium phosphate was added to raise the pH to 6.8 to simulate intestinal conditions for the rest of the release period while maintaining sink conditions [25]. A weight of VP-loaded beads—equivalent to 20 mg of VP—was placed in the dissolution medium kept at 37 °C with a rotation speed of 50 rpm [1]. Three mL samples were withdrawn at predefined time points for 8 h duration (0.25, 0.5, 1, 1.5, 2, 3, 4, 6, 8 h) and were replaced with an equal volume of fresh medium. VP concentration in each sample was quantitatively determined using a UV spectrophotometer at 278 nm. Three more samples at 10, 12 and 24 h were withdrawn when VP-loaded Ch/Kc-PEC-based beads were tested. Isoptin^®^ (80 mg; Abbot Laboratories, Lake Bluff, IL, USA) bisects tablet was used as a reference tablet for in vitro release studies. VP dissolution from Isoptin^®^ was evaluated from the tablet as a whole (80 mg) and upon dividing it into four quarters to assure the similarity in the dissolution behavior when the tablet is divided and suspended before oral administration to rabbits, as described under in vivo studies. Results showed that the dissolution profiles of Isoptin whole tablet compared to the divided tablet were highly similar, and the calculated similarity factor (F2) was found to be 69.58. All in vitro drug release studies were performed in duplicate.

The release kinetics were studied by fitting the release profile to zero-order, first-order and Higuchi diffusion model [26]. The time required for the release of 90% of the drug from each system (t_90%_) was calculated and statistically analyzed.

### 2.6. Characterization of the Optimized Systems

#### 2.6.1. Swelling Test

The swelling behavior of two optimized systems (F8 and F8/3.58) obtained from both statistical designs were examined in three different dissolution media: 0.1 N HCl (pH 1.2), phosphate buffer (pH 6.8) and pH-change medium [4,14]. For the pH-change medium, beads were immersed in 0.1 N HCl for the first 2 h, then the medium pH was increased, as described under In Vitro Release Studies, for another 22 h. Beads were first weighed and then immersed in each one of the dissolution media for 24 h at 37 °C. At specified time points (0.5, 1, 2, 4, 6 and 24 h), beads were collected and re-weighed after removal of excess solvent, and the swelling degree was estimated from the following equation,

(3) $$\%\;\mathrm{swelling}=\frac{w_{t}-w_{0}}{w_{0}}\times 100$$

where w_0_ and w_t_, represent the initial weight of the beads and the weight of the swollen beads at each time point, respectively. All experiments were performed in duplicate [27].

#### 2.6.2. Scanning Electron Microscopy (SEM)

The size, surface structure and topography of optimized F8 and F8/3.58 beads were observed using SEM (Quanta 250 FEG; FEI Company, Eindhoven, The Netherlands). The beads were mounted on metal grids and sprayed with gold (Emitech K550X sputter coater; Quorum Technologies, Laughton, East Sussex, UK), and photomicrographs were taken.

#### 2.6.3. Fourier Transform Infrared (FTIR) Spectroscopy

FTIR scanning was performed for VP, Ch, Kc, drug free-Ch beads, drug-free Ch/Kc-PEC beads in addition to F8 and F8/3.58 beads. 2–3 mg of each sample was ground then mixed with 100 mg of potassium bromide, compressed into thin discs using a hydrostatic press and finally scanned with FTIR spectrophotometer (IR Affinity-1; Shimadzu, Kyoto, Japan) over wavelength range 4000–400 cm^−1^.

### 2.7. In Vivo Pharmacokinetic Study

#### 2.7.1. Study Design

In vivo absorption studies were carried out in rabbits to compare the PK profiles of VP from F8/3.58 beads and Isoptin^®^ tablets following oral administration of single doses equivalent to 10 mg/kg VP each [28]. Male albino rabbits (body weight; 2.0–2.5 kg) were assigned randomly into two treatment groups of six rabbits each using a non-blind, two-treatment, two-period, randomized crossover design with 7 days washout period. Rabbits were housed according to National Institute of Health guidelines, and the study protocol was approved by Research Ethics Committee (REC) for experimental and clinical studies at Faculty of Pharmacy, Cairo University, Cairo, Egypt (PI (1343, 30/10/2017)). The rabbits were supplied by the Laboratory Animal Center at Faculty of Pharmacy, Cairo University, Egypt. Each rabbit was housed individually and allowed free access to food and water for the duration of the experiment.

#### 2.7.2. Drug Administration and Dosing

F8/3.58 beads equivalent to the administered doses were filled into size “2” hard gelatin capsules, which were administered to the rabbits orally followed by water to ensure swallowing. The capsule acted only as a reservoir to contain the formulated beads. The capsule shell readily dissolved in dissolution media within a few minutes as observed in in vitro release studies. Similarly, Isoptin bisects tablets were first divided, and then an amount equivalent to 10 mg/kg was dispersed in water by sonication immediately before administration. A volume of the suspensions equivalent to the calculated dose was given to the animals through the oral route.

#### 2.7.3. Blood Sampling

Blood samples (3 mL) were withdrawn from the ear vein of each rabbit at predetermined time intervals: 0 (pre-dose), 0.5, 1, 1.5, 2, 3, 4, 5, 6, 8, 12 and 24 h post-administration of a treatment into heparinized glass tubes. The plasma was immediately separated from the blood cells by centrifugation at 4000 rpm for 15 min and stored frozen until analysis by liquid chromatography-mass spectrometry (LC-MS/MS).

#### 2.7.4. Sample Preparation and LC-MS/MS Analysis

Plasma concentrations of VP were determined using a selective, sensitive and accurate LC-MS/MS method that was developed and validated before use [24]. Briefly, VP was analyzed in plasma samples using Triple Quadrupole LC-MS/MS Mass Spectrometer (AB Sciex Instruments, Framingham, MA, USA). One hundred µL of the internal standard stock solution (100 ng/mL of torsemide) was added to plasma samples (0.5 mL) and vortexed. Extraction solvent (4 mL ethyl acetate) was added to the samples, and the mixtures were vortexed for 1 min then centrifuged for 10 min at 4000 rpm and 4 °C (Nüve NF815; Ankara, Turkey). The organic layer was separated from each sample and dried using vacuum concentrator (Eppendorf 5301; Hamburg, Germany). The dried residues were reconstituted with 100 µL of the mobile phase (acetonitrile: 0.1% formic acid in water (80:20, *v*/*v*)) and finally transferred to the auto-sampler vials, where 10 µL was injected into the LC-MS/MS.

#### 2.7.5. Pharmacokinetic Analysis

The plasma concentration-time data were analyzed via non-compartmental PK model using Kinetica 2000 software (version 3.0, Philadelphia, PA, USA) and PK parameters of VP post-administration of either F8/3.58 beads or Isoptin^®^ tablets were estimated. The observed maximum plasma concentration (*C_max_*) and time to reach *C_max_* (*T_max_*) were estimated directly from the plasma concentration-time profile. Elimination rate constant (*k*) was estimated from the terminal elimination line using the log-linear regression analysis, and the half-life (*t*_1/2_) was calculated as *t*_1/2_ = 0.693/*k*. The area under the curve (AUC_0-*t*_) was calculated using the trapezoidal rule from zero time to the last time of blood sample and the area under the curve from zero to infinity (AUC_0-∞_) was calculated as (AUC_0-∞_) = (AUC_0-*t*_) + C*_t_*/*k*, where *C_t_* is the last measured concentration at time *t*. The mean residence time (MRT) was calculated from AUMC_0-∞_/AUC_0-∞_, where *AUMC*_0-∞_ is the area under the first moment curve. In addition, half value duration (HVD) was calculated and used to measure retard quotient (R_D_). R_D_ is a parameter that gives the factor by which the half value duration (HVD) is extended in the sustained release (SR) formulation compared to the immediate release (IR) one, where it is calculated as the ratio between the HVD of SR system relative to IR system. A R_D_ value of 1, 1.5, 2 and 3 indicates no, low, medium and strong retardation, respectively, of the SR system in vivo [29].

### 2.8. Statistical Analysis

All in vitro data points are the average of at least two independent experiments performed, and the values are expressed as means ± SD. Statistical significance of the experiments’ results was assessed by Student’s *t*-test (two-tailed, *p* < 0.05). For in vivo studies, statistical inferences were based on untransformed values for *C_max_* and AUC variables and observed values for *t*_1/2_. The nonparametric Signed Rank Test (Mann–Whitney’s test) was used to compare *T_max_* between the two treatment groups. The one-way analysis of variance (ANOVA) *F*-test was used for testing the equality of several means. For multiple comparison, the procedure used was the least significant difference (LSD). Statistical analyses were carried out using SPSS (SPSS^®^ Statistics software program, version 17.0, International Business Machines Corp., Armonk, NY, USA) or Design-Expert^®^ software (version 7.0.0, Stat-Ease Inc., Minneapolis, MN, USA).

## 3. Results and Discussion

### 3.1. Characterization of Superhydrophobic Substrates

**Figure 2:**
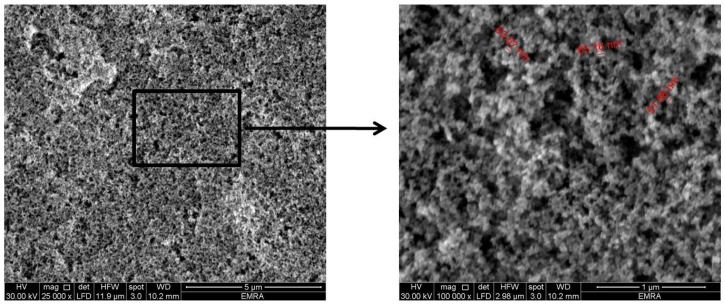
SEM images showing the nano-length hierarchical structure of the prepared superhydrophobic substrate (magnification power 25,000× and 100,000×).

Following application of “NeverWet^®^” spray on the glass slides, a rough structure was created on the surface, as shown in SEM images of Figure 2. It is clear that the glass surface shows a hierarchical structure in the nano-metric range (50–65 nm), whose function is to trap air bubbles in the small scale pockets at the solid liquid interface leading to a composite interface with lower surface wettability [30].

**Figure 3:**
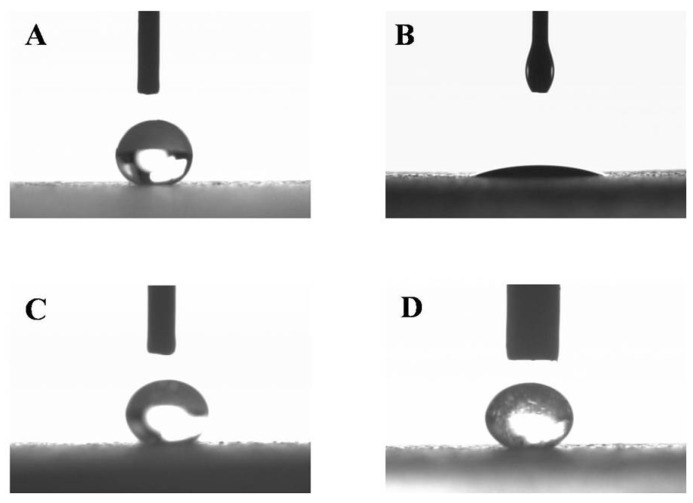
Images of contact angles for (**A**) water, (**B**) olive oil, (**C**) optimized VP-loaded Ch-based dispersion F8 and (**D**) optimized VP-loaded Ch/Kc-PEC-based dispersion F8/3.58.

When a water droplet was dropped on the surface of the treated glass, it showed a significantly higher CA (>150°) with lower SA (<10°) compared to untreated smooth glass surface (Table 3 and Figure 3A). The surface still offered good superhydrophobicity for both optimized VP-Ch-hydrogel (F8) and VP-Ch/Kc-PEC-hydrogel (F8/3.58) dispersions, as indicated by the measured CA (Figure 3C,D). A slight reduction in the CA (146.57° ± 2.45°) and increment in the SA (11.67° ± 1.53°) were observed with VP-Ch hydrogel system (F8) when compared to water (151.03° ± 1.08° and 1.00° ± 0.00°, respectively), which could be attributed to the viscous and sticky nature of Ch. On the other hand, when a droplet of oil was dropped on the surface, it showed complete spreading and wetting of the surface (Figure 3B), which was confirmed by the measured CA value of 23.62° ± 1.02° indicating that the surface has hydrophobic activity with no oleophobic one.

The results presented in Table 3 demonstrate that air entrapped in the air pockets at the surface/liquid interface is the main reason for stopping the liquid from spreading and wetting the surface [22].

### 3.2. Formulation of Ch- and Ch/Kc-Based Beads

Ch is a cationic polysaccharide, which is soluble in acidic solution. It tends to harden into hydrogel upon covalent cross-linking with glutaraldehyde, glycol or glyoxal solution [11,12]. In addition, Ch has the ability to form polyelectrolyte complexes (PEC) with various anionic polyelectrolytes (e.g., carrageenan) through strong electrostatic interaction. These PEC tend to modify the surface properties of the formed beads and prolong the release of encapsulated drug [11,14]. In this work, Ch beads were formed on superhydrophobic surfaces by in situ cross-linking in a glutaraldehyde-saturated atmosphere instead of using glutaraldehyde solution to prevent drug loss and achieve the highest possible EE%. The use of glutaraldehyde-saturated atmosphere also excludes the need to remove excess glutaraldehyde solution [4]. The effect of Ch MW and its concentration on the EE% and t_90%_ were statistically analyzed in a 3^2^ full factorial design. Afterwards, the selected system from the first design was complexed with Kc to formulate PEC beads, which were then cross-linked with glutaraldehyde, as described before, to further control the release of VP from the formed beads. The effect of Kc concentration was studied through a response surface design to further select the optimized system.

### 3.3. Evaluation of VP-Loaded Ch-Based Beads

#### 3.3.1. Encapsulation Efficiency (EE%)

**Figure 4:**
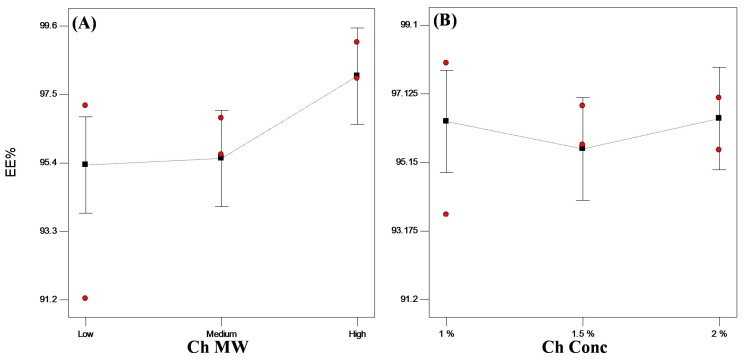
Line plot for the main effects of (**A**) Ch molecular weight (MW) and (**B**) Ch concentration on the encapsulation efficiency (EE%) of VP-loaded Ch-based beads.

In general, encapsulation of hydrophilic drugs into spherical systems is challenging and problematic due to the loss of drug to the external phase [1,5,31]. A trial was done to prepare VP-loaded Ch hydrogel with conventional ionotropic gelation technique [32], and only 27.80% of VP was encapsulated. In this work, bead solidification took place on the prepared superhydrophobic surfaces in a glutaraldehyde atmosphere without any contact with the glutaraldehyde solution, thus achieving higher EE% reaching about 94% *w*/*w* (Table 1). Upon statistical analysis of the data, only Ch MW significantly affected (*p* < 0.05) the encapsulation of the drug into the beads, whereas Ch concentration did not show any significant effect (*p* > 0.05). High MW Ch showed significantly higher (*p* < 0.05) EE% than lower MW Ch (Figure 4). This might be attributed to the difference in viscosity of the prepared dispersions as high MW Ch yields more viscous dispersions than lower or medium MW Ch. The more viscous dispersion consequently hinders the diffusion of the drug to the external superhydrophobic surface, therefore ensuring beads with ultrahigh EE% (>98% *w*/*w*) [3,33].

#### 3.3.2. In Vitro Release Studies

Evaluation of the in vitro rate at which the drug is released from a delivery system is important to determine the release mechanism as well as to predict in vivo release upon oral administration [34]. In general, several factors affect drug release from Ch hydrogel beads, such as extent of cross-linking within the polymer, polymer composition, morphology, size and density of the beads as well as the physicochemical properties of the incorporated drug. Drug release may occur via desorption, diffusion through swollen beads, erosion of the beads or by a combined diffusion/erosion mechanism [12]. In case of hydrophilic matrices, the diffusion process is the main controlling release mechanism for hydrophilic drugs, whereas erosion process is predominant for hydrophobic ones [35].

In order to evaluate VP release from the prepared Ch systems, the beads were suspended in 0.1 N HCl followed by phosphate buffer, as previously described. All the prepared systems showed a high initial burst release ranging from 35% to 55% *w*/*w* followed by a slower rate of release. This burst effect is common with hydrophilic drugs, where a rapid initial release of the drug for a short time occurs upon contact with the dissolution medium, followed by the desired release rate [36]. Hydrophilic drugs tend to form microcavities in the gel layer which promote drug diffusivity through the swollen gel layer, hence its initial rapid release [37]. In addition to its hydrophilic nature, VP, being an acidic salt of a weak base with pKa value of 8.6, was rapidly ionized and solvated in acidic medium (0.1 N HCl), resulting in high initial burst effect and a faster release rate compared to phosphate buffer [38,39]. Unentrapped drug adhered to the surface of the beads could also contribute to the initial burst release. An initial rapid release of a fraction of the dose followed by sustained drug release for a specified time can be beneficial when a quick onset of action is desired first, followed by a sustained drug action. Alternatively, release of VP in acidic medium can be prevented by coating the beads with an enteric coat.

Kinetic analysis of the release data showed that all formulated systems followed Higuchi diffusion model [12], where penetration of the dissolution medium inside the beads followed by the dissolution of the drug and its diffusion through the gel layer are the main factors affecting drug release [40,41].

**Figure 5:**
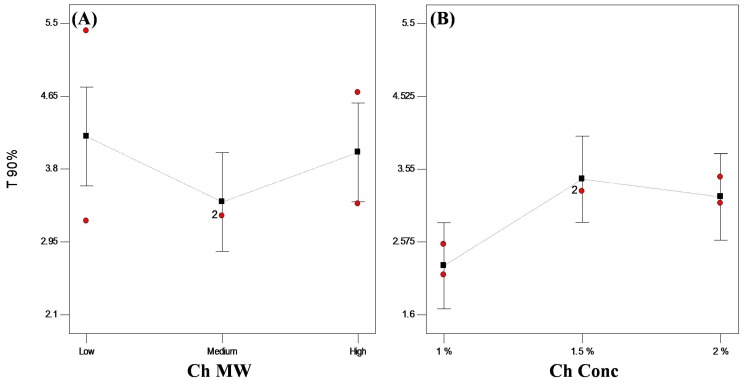
Plot for the main effects of (**A**) Ch MW and (**B**) Ch concentration on t_90%_ of VP from VP-loaded Ch-based beads.

Based on VP release mechanism, t_90%_ was calculated from the best-fitted equation (Table 1). Statistical analysis revealed that t_90%_ was significantly (*p* < 0.05) affected by Ch concentration. On the other hand, changing Ch MW did not have a significant (*p* > 0.05) effect on the release rate. Similar findings were observed by Yassin et al. [1], who found that the in vitro release of VP did not depend on Ch MW. It was observed that increasing Ch concentration from 1.0% to 1.5% *w*/*v* resulted in a slower drug release with higher t_90%_ value (Figure 5). Increasing the polymer concentration results in more cross-linking within the polymer network and increases the hydrogel tortuosity, which convolutes the diffusional path and subsequently reduces drug diffusion within the beads [38,42,43].

However, further increase of Ch concentration from 1.5% to 2.0% *w*/*v* did not prolong the t_90%_ value. This could be justified in terms of the percolation theory, where discontinuity of certain property of a system exists above a critical point (percolation threshold). According to this theory, increasing polymer concentration above a certain percolation threshold will no longer slow down the drug release because the gel layer formed above this concentration is coherent and homogenous, thus controlling the hydration of the system [44]. Similar results were reported where the increase in HPMC concentration above 10% did not show any further reduction in metronidazole dissolution rate irrespective to the viscosity grade [33].

#### 3.3.3. Statistical Optimization of VP-Loaded Ch-Based Beads

Optimization results revealed that system “F8” is the optimized system when the optimization conditions were set to the highest EE% and longest t_90%_ with desirability factor of 0.703. F8 showed 98.54% EE (*w*/*w*) and t_90%_ of 4.04 h. The optimized system (F8), which is composed of 1.5% *w*/*v* high MW Ch, was integrated into another design to form PEC with Kc to modulate the sustainability of VP release.

### 3.4. Evaluation of VP-Loaded Ch/Kc-PEC-Based Beads

#### 3.4.1. Encapsulation Efficiency (EE%)

**Figure 6:**
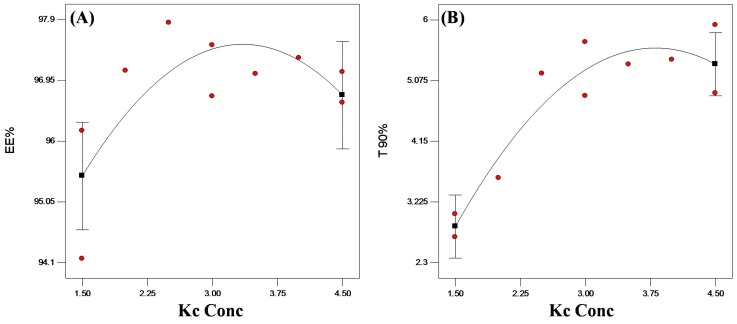
Statistical plot for the main effects of Kc concentration on (**A**) EE% and (**B**) t_90%_ of VP from VP-loaded Ch/Kc-PEC-based beads.

Table 2 shows the EE% of all PEC formulated systems. All systems demonstrated EE higher than 95% *w*/*w* of VP. Upon statistical analysis of the data, Kc concentration did not show any significant (*p* > 0.05) effect on the encapsulation of VP into PEC beads (Figure 6A).

#### 3.4.2. In Vitro Release Studies

Aiming to further control VP release from the formulated beads, Kc was added to Ch to produce VP-loaded Ch/Kc-PEC-based beads. Upon kinetic analysis of the release data, all the formulated beads still followed Higuchi diffusion model. The t_90%_ values of the prepared beads were calculated from the best-fitted equation and are presented in Table 2. The addition of Kc in increasing concentrations up to 3.5% *w*/*v* resulted in significantly (*p* < 0.05) longer t_90%_ reaching 5.32 ± 0.65 h compared to 2.87 ± 0.24 h upon using 1.5% *w*/*v* (Figure 6B). This retardation in VP release was due to complex formation between the positively charged Ch and the negatively charged Kc, which reduced drug diffusion from the beads [13,14]. At a concentration higher than 3.5% *w*/*v*, no further reduction in VP release rate was observed, which might be due to complete consumption of the free amino groups in Ch molecules. In general, Ch is a linear polysaccharide composed of linked D-glucosamine monomers (i.e., each monomer has 1−NH_2_ group that changes to −NH_3_^+^ upon protonation), whereas Kc is a linear polysaccharide with one sulfate group (−OSO_3_^−^) per each disaccharide moiety. Thus, each monosaccharide of Ch (C_6_H_11_O_4_N with monomer wt. 161g/monosaccharide) will react with a disaccharide moiety of Kc (C_12_H_18_0_12_S with dimer wt. 386g/disaccharide) to produce the PEC. By calculation, it was found that 1.5 g of Ch will complex with almost 3.59 g of Kc. Therefore, at Kc concentration higher than 3.59% *w*/*v*, there is no more complex formation and subsequently, no further retardation in drug release.

#### 3.4.3. Statistical Optimization of VP-Loaded Ch/Kc-PEC-Based Beads

Statistical analysis of the effect of Kc concentration showed that the optimized system is composed of 3.58% *w*/*v* of Kc when combined with system F8 previously selected from the first statistical design. This choice confirms the above suggestion that one monosaccharide of Ch interacts with a disaccharide of Kc, as previously discussed. The desirability factor of the suggested optimized system (F8/3.58) was 0.892 after setting the optimization conditions at the highest EE% and longest t_90%_. F8/3.58 beads showed an EE% of 91.99 ± 0.24% *w*/*w* and Higuchi diffusion model of release with t_90%_ of 5.36 ± 0.40 h (Table 2).

**Figure 7:**
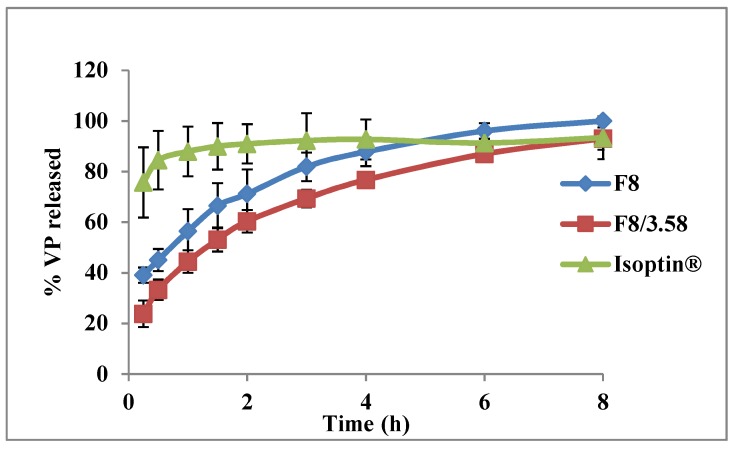
Release profiles of VP from the optimized systems compared to the marketed product (Isoptin^®^).

The in vitro release profiles of the two optimized systems from both designs (F8 and F8/3.58) and the marketed VP tablets (Isoptin^®^) are graphically illustrated in Figure 7. The release profile of F8/3.58 beads showed a more controlled release with less initial burst effect (23.82% *w*/*w*) compared to F8 beads.

### 3.5. Characterization of Optimized PEC Beads

#### 3.5.1. Swelling Test

**Figure 8:**
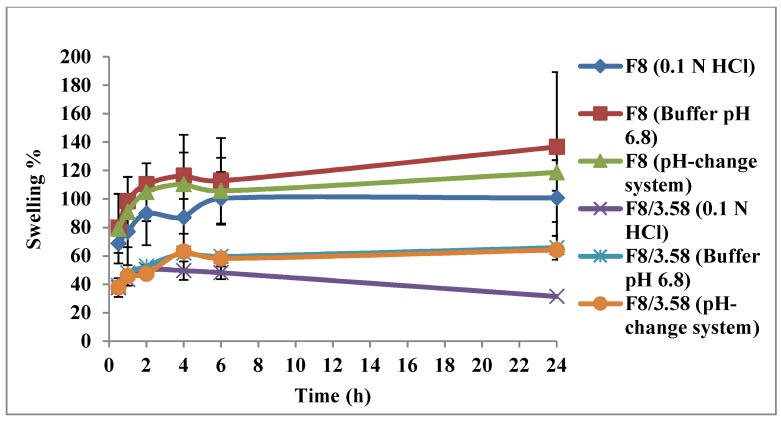
Swelling profiles of the optimized systems F8 and F8/3.58 in different media; 0.1 N HCl (pH 1.2), phosphate buffer (pH 6.8) and pH-change system (HCl for the first 2 h then phosphate buffer for 22 h).

The swelling behavior of F8 and F8/3.58 beads was studied in different media for 24 h. The results are graphically illustrated in Figure 8. All beads showed pH-sensitive swelling behavior where the degree of swelling was lower at acidic pH (1.2) and increased upon incubating the beads in phosphate buffer (pH = 6.8). F8/3.58 beads showed a lower degree of swelling compared to F8 beads at all pH values, which could be attributed to the strong ionic interaction between positively charged Ch and negatively charged Kc. This strong interaction hinders the penetration of water into the beads, thus less swelling and less drug diffusion to the external phase result in slower drug release rate. At acidic pH, the amino groups of Ch are protonated, thereby increasing the charge density, which interacts strongly with the acidic sulfonic group of Kc, producing stronger complex with a reduced degree of swelling. At higher pH, deprotonation of the amino groups occurs, which weakens the extent of ionic interactions and increases the degree of swelling [13].

#### 3.5.2. Scanning Electron Microscope

**Figure 9:**
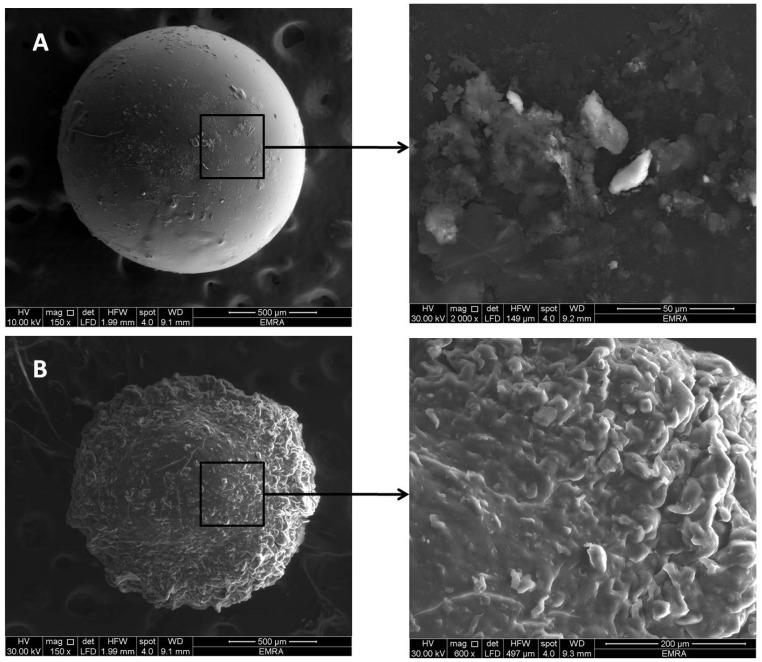
Images of (**A**) F8 Ch-based beads and (**B**) F8/3.58 Ch/Kc-PEC-based beads.

SEM images of F8 beads showed smooth spherical shape with average size of 1.43 ± 0.07 mm (Figure 9A). Some clusters of the drug appear deposited on the surface, presenting surface drug that may have contributed to the observed initial burst release, as described under In Vitro Release Studies. F8/3.58 PEC-based beads showed spherical spheres with a rough surface full of folds with average bead size of 1.24 ± 0.16 mm. The smaller average size of PEC-based beads relative to Ch-based beads could be the result of the strong ionic interaction between Ch and Kc in the formed complex. These folds may act as an additional barrier to the diffusion of the drug to an external phase, explaining the more controlled VP release associated with PEC-based beads (Figure 9B).

#### 3.5.3. Fourier Transform Infrared (FTIR) Spectroscopy

**Figure 10:**
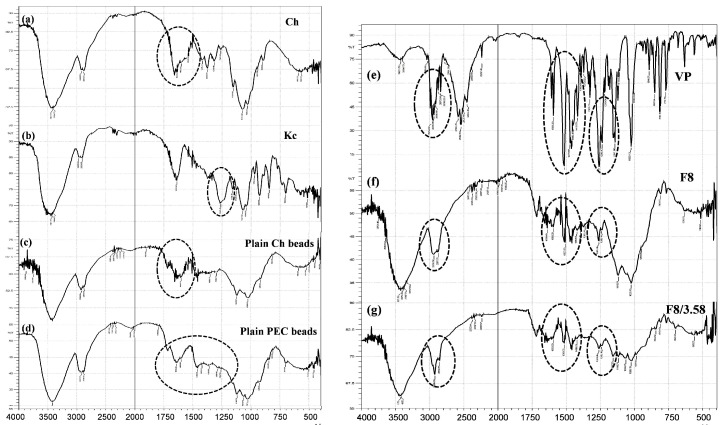
Spectra of (**a**) Ch, (**b**) Kc, (**c**) plain Ch-beads, (**d**) plain PEC beads, (**e**) VP, (**f**) VP-loaded Ch-based beads (F8) and (**g**) VP-loaded Ch/Kc-PEC-based beads (F8/3.58). Dotted circles indicate locations with characteristic peaks.

FTIR spectroscopy was employed for the detection of any chemical interaction among the formulation components and VP in the prepared beads by pointing out the characteristic molecular groups [45]. Figure 10 shows the IR spectra of Ch, Kc, plain (drug-free) Ch-beads, plain PEC-beads, VP, VP-loaded F8 beads and VP-loaded F8/3.58 beads. The IR spectrum of Ch presented in Figure 10a showed several characteristic peaks at different locations including vibrations of the primary amine groups at 1631 cm^−1^ [46,47]. The IR spectrum of Kc (Figure 10b) showed characteristic significant peak at 1261 cm^−1^ corresponding to the (−OSO_3_^−^) stretching vibrations of Kc [48]. Plain Ch-beads showed all of the characteristic peaks of Ch at the same position (Figure 10c). On the other hand, plain PEC beads (Figure 10d) revealed the disappearance of the characteristic (−OSO_3_^−^) vibrations at 1261 cm^−1^ of Kc, which could be considered as evidence to the inclusion of Kc in PEC formation [13]. In addition, the peak assigned to the amine band of Ch at 1631 cm^−1^ was slightly shifted, indicating that the amine group was protonated to a NH_3_^+^ group in the complex [16]. Therefore, it could be deduced that the Ch/Kc PEC was formed by an electrostatic interaction between the (−OSO_3_^−^) group of Kc and the NH_3_^+^ group of Ch.

VP spectrum (Figure 10e) showed C–H stretching peaks of methylene and methoxy groups at (2954–2839 cm^−1^), a sharp characteristic peak of C≡N at 2237 cm^−1^, C–H stretching of the benzene ring at 1593, 1516 and 1473 cm^−1^ and a strong C–O stretching vibrations of the aromatic ethers at 1261 cm^−1^ [13,49].

Figure 10f,g, representing VP-loaded F8 beads and F8/3.58 beads respectively, demonstrate attenuated peaks of VP that might be due to drug dilution in the systems. However, there was no shift in the position of the drug characteristic peaks, which indicates the lack of any significant chemical interaction between VP and the formulation constituents.

### 3.6. In Vivo Pharmacokinetic Study

The in vivo behavior of capsules filled with F8/3.58 beads were assessed and compared to Isoptin^®^ tablets by monitoring VP plasma levels for 24 h post-oral administration to rabbits in a crossover design. The rabbit model was reported to be more suitable than dogs, rats and mice to explain VP clinical PK and drug interactions, which take place in rabbits in patterns very similar to those found in humans [50].

**Figure 11:**
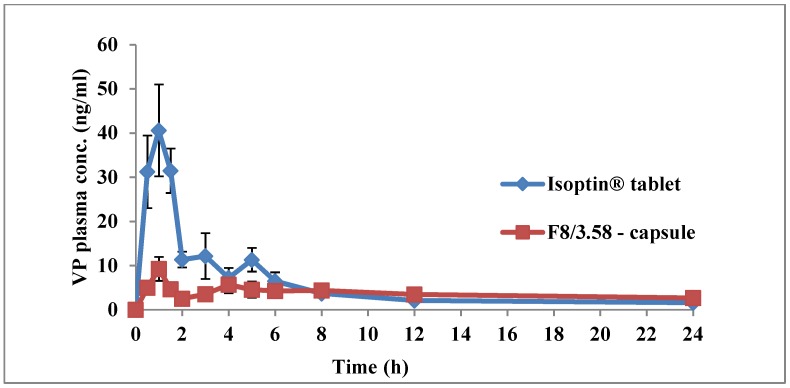
Mean (±SD) plasma VP concentrations following oral administration of Isoptin^®^ tablets and F8/3.58 beads filled capsules to six albino rabbits.

Plasma concentration-time curves of both F8/3.58 beads and Isoptin tablets are shown in Figure 11. The plasma profile of the marketed product showed a significantly (*p* < 0.05) higher plasma concentration up to 6 h post-administration, followed by fast elimination and rapid decline in drug concentration in the subsequent time intervals. On the other hand, F8/3.58 beads exhibited a slowly declining curve that maintained VP plasma concentration for a prolonged time.

The mean PK parameters (*n* = 6) following the two treatments are summarized in Table 4. *T_max_*, *k*, *t*_1/2_, AUC_0-t_ and AUC_0-∞_ estimates from both treatments were not statistically significantly different (*p* > 0.05). The insignificant difference between the values of both AUC_0-t_ and AUC_0-∞_ is an indication of the absence of any decline in VP bioavailability from the developed sustained release formulation, although suffering from an extensive first-pass effect [51]. The significantly higher mean *C_max_* estimate observed from the marketed product (41.78 ± 21.49 ng/mL) compared to the mean estimate from F8/3.58 beads (10.41 ± 6.97 ng/mL) was expected due to the rapid and immediate release of VP from Isoptin, thus achieving a high plasma concentration. Table 4 demonstrates that the mean MRT estimate obtained from F8/3.58 beads (25.19 ± 8.85 h) was 121% larger and statistically significantly higher relative to the mean estimate obtained from the marketed product (11.38 ± 2.29 h). The rapid decline in the plasma level due to rapid elimination from the body is typical of conventional IR products. The significantly higher (*p* < 0.05) MRT obtained from the F8/3.58 beads indicates a longer residence of the drug molecules in the body, confirming that the formulated beads offered a sustained release behavior in vivo, as previously observed in vitro. This result was further confirmed by the significantly higher HVD values calculated (*p* < 0.05) from SR F8/3.58 beads compared to IR Isoptin. The calculated R_D_ ratio showed 5.24 times extension in the HVD, suggesting a strong retardation (R_D_ > 3) of the formulated SR beads in vivo [29]. It was noticed that VP plasma concentrations from F8/3.58 beads after 8 h were only slightly higher compared to Isoptin. This may indicate that the in vivo drug release from the beads was much slower compared to the observed in vitro drug release, which is usually encountered when testing controlled-release formulations in different animal models. Higher VP plasma concentrations can be achieved by increasing the rate of drug release from the beads in vivo, which may lead to an increase in the rate and extent of drug absorption, thus resulting in higher VP plasma levels at all time points.

The similar *T_max_* estimates obtained from both treatments could be due to the initial burst release of VP from the beads, as previously described under In Vitro Release studies. Similar results were reported by other researchers during preparation of controlled-release systems where lopinavir-loaded nanoparticles showed 1.3-fold increase in MRT compared to free lopinavir, although exhibiting the same *T_max_* [52]. Similar findings were also reported when rifampicin was loaded into ethyl cellulose-coated nonpareil beads [53].

It was also noted that the variability in plasma concentrations in the six rabbits following administration of F8/3.58 beads was less compared to Isoptin tablets during the absorption phase, which further confirms that controlled-release multiparticulate systems result in reduced inter- and intra-individual variation. Thus, VP-loaded Ch/Kc-PEC-based beads could be a promising cost-effective, scalable SR multiparticulate formulation that can be added to the industrial arsenal to alter the PK profile of VP and similar drugs to ensure more effective therapeutic and clinical applications.

## 4. Conclusions

In this study, we demonstrated that ultrahigh VP-loaded Ch/Kc-PEC-based beads prepared on superhydrophobic substrates are promising formulations that could control the release of VP and extend its duration of action in vivo. The results obtained in this study suggest that the developed multiparticulate F8/3.58 system may be an alternative to conventional immediate-release VP tablets, which necessitate frequent administration. The proposed formulation is an easily modifiable delivery platform for ultrahigh encapsulation of water-soluble therapeutic molecules, the release profiles of which are usually difficult to control via conventional encapsulation techniques.

## Figures and Tables

**Table 1:** Composition of VP-loaded Ch beads, independent variables and measured responses of the 3^2^ full factorial experimental design.

System #	Ch MW	Ch % (*w*/*v*)	EE %	t_90%_ (h)
F1	Low	1	97.00 ± 0.97	2.75 ± 0.03
F2		1.5	94.19 ± 4.19	4.31 ± 1.56
F3		2	96.49 ± 2.53	4.09 ± 1.53
F4	Medium	1	95.82 ± 3.08	2.33 ± 0.29
F5		1.5	96.21 ± 0.79	3.25 ± 0.00
F6		2	96.25 ± 1.06	3.26 ± 0.25
F7	High	1	98.49 ± 0.48	3.02 ± 0.18
F8		1.5	98.54 ± 0.78	4.04 ± 0.92
F9		2	98.82 ± 0.31	3.52 ± 0.59

Data are mean value ± SD (*n* = 2).

**Table 2:** Composition of VP-loaded polyelectrolyte complex (PEC) beads and the measured responses of the one factor design.

System #	High MW Ch % (*w*/*v*)	Kc % (*w*/*v*)	EE % (*w*/*w*)	t_90%_ (h)
F8/1.50	1.5	1.5	95.16 ± 1.41	2.87 ± 0.24
F8/2.00	1.5	2	97.10 ± 0.85	3.59 ± 0.01
F8/2.50	1.5	2.5	97.85 ± 0.07	5.19 ± 0.84
F8/3.00	1.5	3	97.1 ± 0.57	5.25 ± 0.58
F8/3.50	1.5	3.5	97.05 ± 0.49	5.32 ± 0.65
F8/4.00	15	4	97.3 ± 1.41	5.39 ± 0.69
F8/4.50	1.5	4.5	96.84 ± 0.34	5.40 ± 0.24
*F8/3.58	1.5	3.58	91.99 ± 0.24	5.36 ± 0.40

Data are mean value ± SD (*n* = 2). * The optimized system.

**Table 3:** Static contact angles (CA), sliding angles (SA) and percentage trapped air for the tested liquids on superhydrophobic treated glass surfaces compared to their CA on untreated glass surfaces.

System	Untreated Surface	NeverWet^®^-Treated Surface				
	CA (°)	CA (°)	SA (°)	f	1-f	% Trapped Air
Water	46.7 ± 0.85	151.03 ± 1.08	1.00 ± 0.00	0.0740	0.9258	92.58
Olive Oil	29.70 ± 4.19	23.62 ± 1.02	-	1.0250	-	-
F8	56.40 ± 6.78	146.57 ± 2.45	11.67 ± 1.53	0.1065	0.8934	89.34
F8/3.58	49.00 ± 3.39	151.40 ± 2.62	4.33 ± 0.58	0.0736	0.9263	92.63

Data are mean value ± SD (*n* = 3).

**Table 4:** Mean pharmacokinetic (PK) parameters of VP following oral administration of Isoptin^®^ and F8/3.58 beads to six albino rabbits.

Parameter	Isoptin	F8/3.58	*p*-Value
C_max_ (ng/mL)	41.78 ± 21.49	10.41 ± 6.97 *^b^*	0.03
AUC_0-∞_ (ng h/mL)	165.39 ± 84.16	148.43 ± 76.92	0.81
AUC_0-24_ (ng h/mL)	138.08 ± 69.10	107.25 ± 44.46	0.24
k (h^−1^)	0.06 ± 0.02	0.05 ± 0.02	0.14
t_1/2_ (h)	11.50 ± 3.26	16.38 ± 6.85	0.13
MRT (h)	11.38 ± 2.29	25.19 ± 8.85 *^b^*	0.03
HVD (h)	1.37± 0.12	7.18 ± 3.39 *^b^*	0.02
T_max_ (h)	1 (1–1.5)	1 (1–6)	0.66

Data are mean value ± SD (*n* = 6). *^b^p* < 0.05 versus Isoptin.
